# Supplementary figures and images for: The NLRP1 inflammasome is an essential and selective mediator of axon pruning in neurons
Source: EMBO Rep. 2025 Feb 26;26(7):1724–36. doi: 10.1038/s44319-025-00402-y (PMC11977209; doi:10.1038/s44319-025-00402-y)

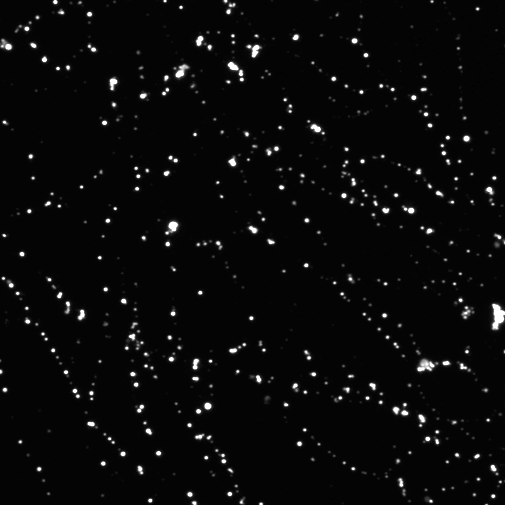

Supplement: Supplementary file 2 — Source data Fig. 1 [file 44319_2025_402_MOESM2_ESM.zip › Figure 1/1B/Global_10uM_crop.tif]

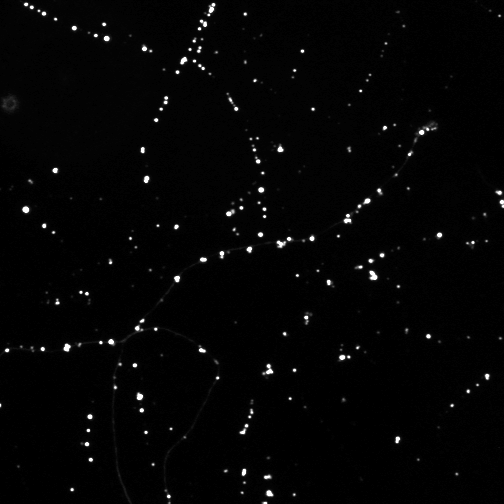

Supplement: Supplementary file 2 — Source data Fig. 1 [file 44319_2025_402_MOESM2_ESM.zip › Figure 1/1B/Global_5uM_crop.tif]

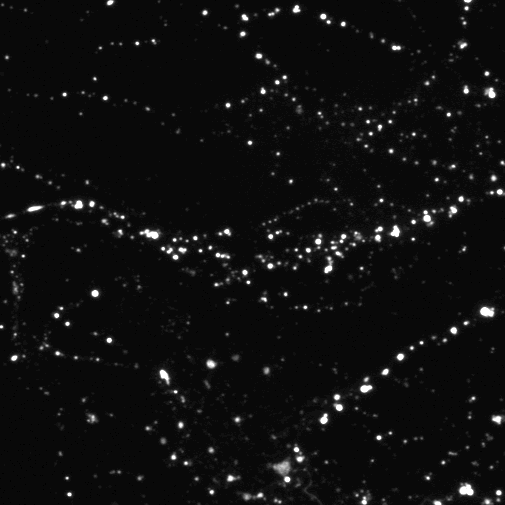

Supplement: Supplementary file 2 — Source data Fig. 1 [file 44319_2025_402_MOESM2_ESM.zip › Figure 1/1B/Global_UNT_crop.tif]

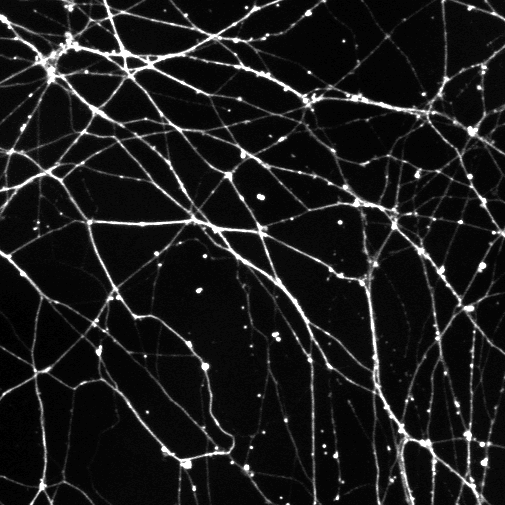

Supplement: Supplementary file 2 — Source data Fig. 1 [file 44319_2025_402_MOESM2_ESM.zip › Figure 1/1B/Local_10uM_crop.tif]

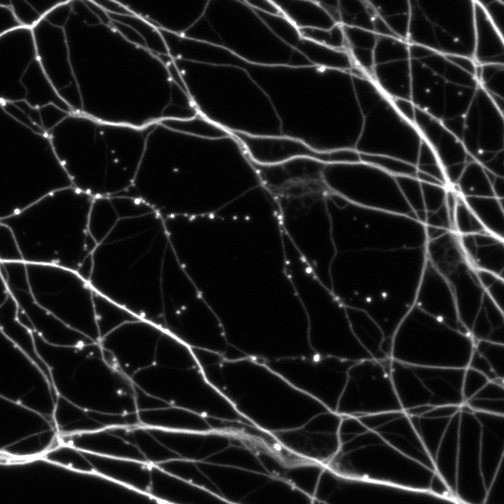

Supplement: Supplementary file 2 — Source data Fig. 1 [file 44319_2025_402_MOESM2_ESM.zip › Figure 1/1B/Local_5uM_crop.tif]

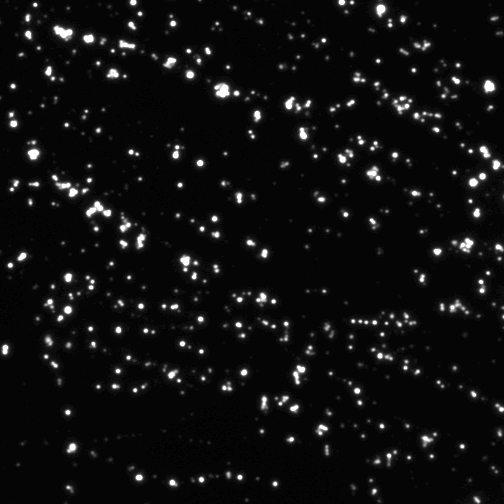

Supplement: Supplementary file 2 — Source data Fig. 1 [file 44319_2025_402_MOESM2_ESM.zip › Figure 1/1B/Local_UNT_crop.tif]

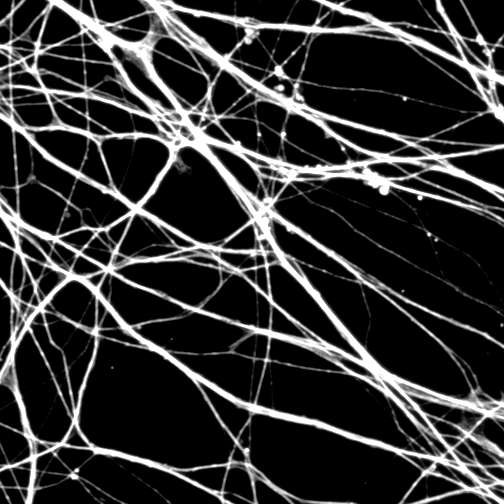

Supplement: Supplementary file 2 — Source data Fig. 1 [file 44319_2025_402_MOESM2_ESM.zip › Figure 1/1B/Maint_10uM_Crop.tif]

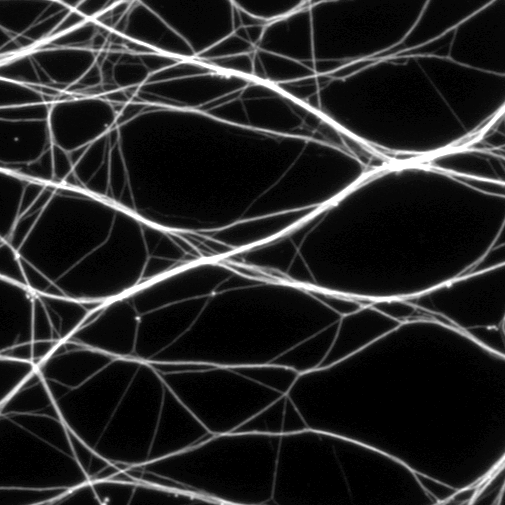

Supplement: Supplementary file 2 — Source data Fig. 1 [file 44319_2025_402_MOESM2_ESM.zip › Figure 1/1B/Maint_5uM_crop.tif]

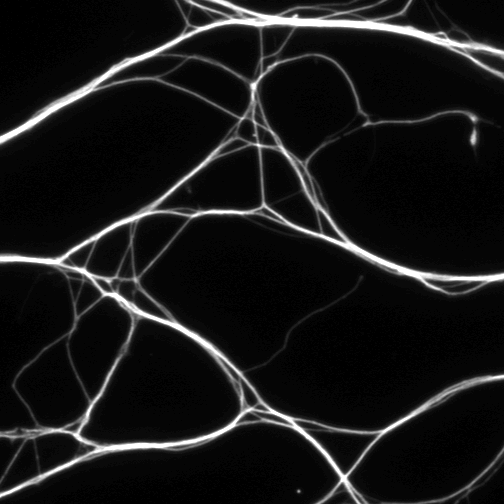

Supplement: Supplementary file 2 — Source data Fig. 1 [file 44319_2025_402_MOESM2_ESM.zip › Figure 1/1B/Maint_UNT_Crop.tif]

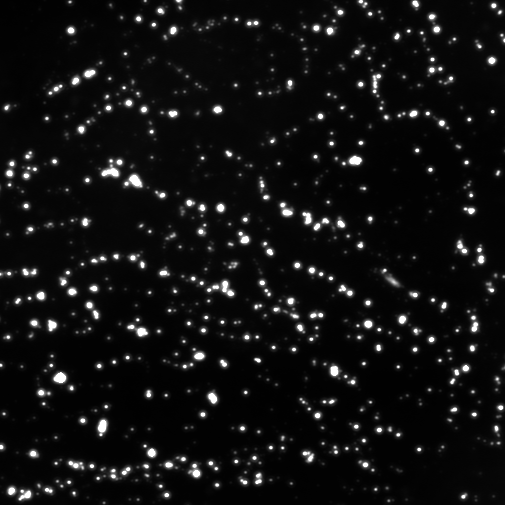

Supplement: Supplementary file 2 — Source data Fig. 1 [file 44319_2025_402_MOESM2_ESM.zip › Figure 1/1D/Casp1-11KO_global_crop.tif]

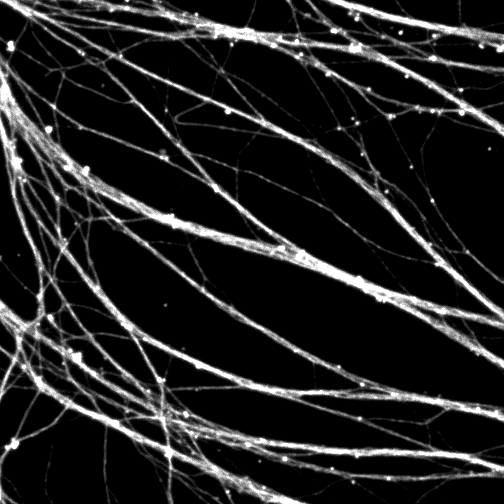

Supplement: Supplementary file 2 — Source data Fig. 1 [file 44319_2025_402_MOESM2_ESM.zip › Figure 1/1D/Casp1-11KO_local_crop.tif]

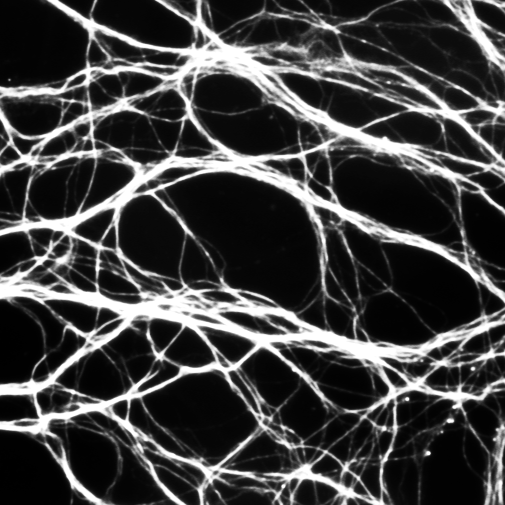

Supplement: Supplementary file 2 — Source data Fig. 1 [file 44319_2025_402_MOESM2_ESM.zip › Figure 1/1D/Casp1-11KO_maint_crop.tif]

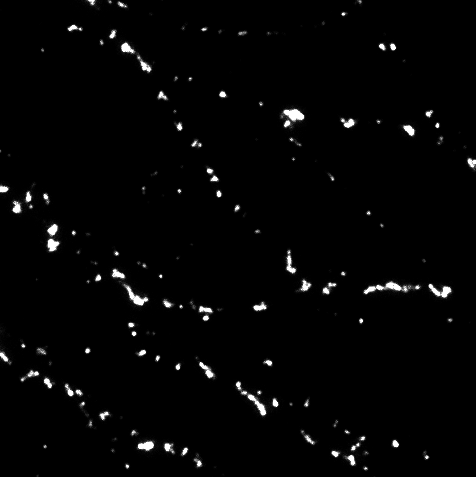

Supplement: Supplementary file 2 — Source data Fig. 1 [file 44319_2025_402_MOESM2_ESM.zip › Figure 1/1D/Casp1SKO_global_crop.tif]

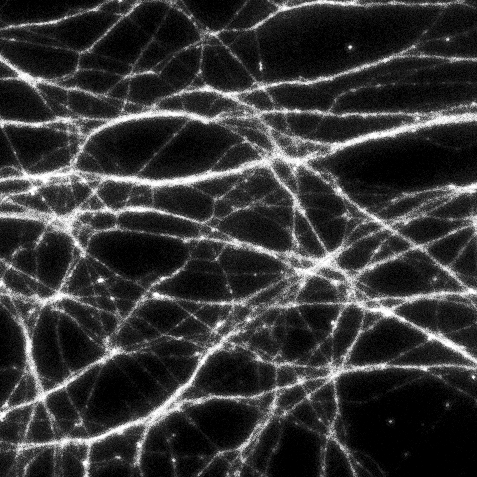

Supplement: Supplementary file 2 — Source data Fig. 1 [file 44319_2025_402_MOESM2_ESM.zip › Figure 1/1D/Casp1SKO_local_crop.tif]

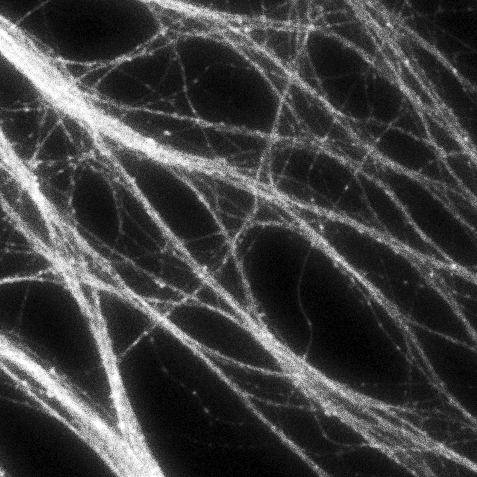

Supplement: Supplementary file 2 — Source data Fig. 1 [file 44319_2025_402_MOESM2_ESM.zip › Figure 1/1D/Casp1SKO_maint_crop.tif]

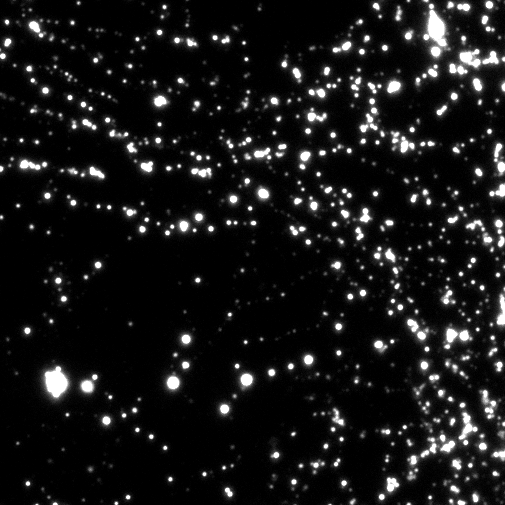

Supplement: Supplementary file 2 — Source data Fig. 1 [file 44319_2025_402_MOESM2_ESM.zip › Figure 1/1D/Global control_crop.tif]

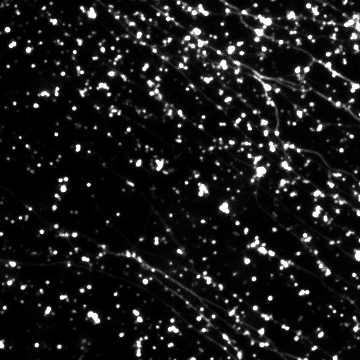

Supplement: Supplementary file 2 — Source data Fig. 1 [file 44319_2025_402_MOESM2_ESM.zip › Figure 1/1D/Local control_crop.tif]

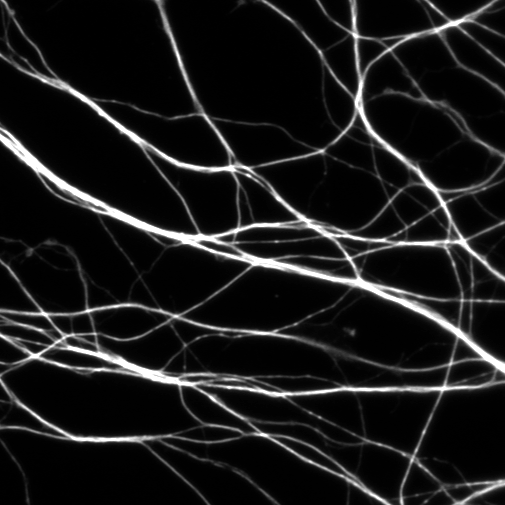

Supplement: Supplementary file 2 — Source data Fig. 1 [file 44319_2025_402_MOESM2_ESM.zip › Figure 1/1D/NGF maint_crop.tif]

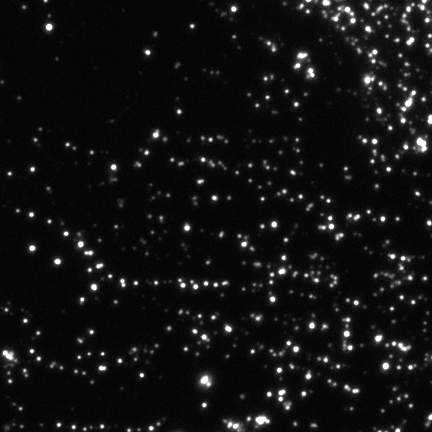

Supplement: Supplementary file 3 — Source data Fig. 2 [file 44319_2025_402_MOESM3_ESM.zip › Figure 2/2C/Aim2_global_crop.tif]

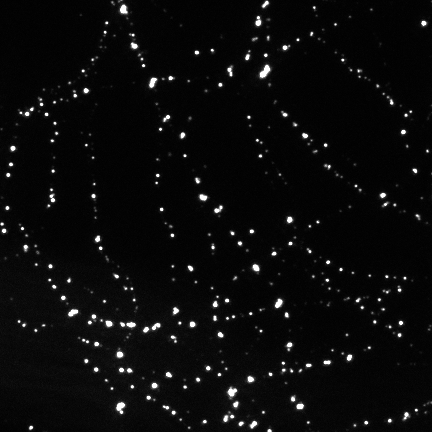

Supplement: Supplementary file 3 — Source data Fig. 2 [file 44319_2025_402_MOESM3_ESM.zip › Figure 2/2C/Aim2_local_crop.tif]

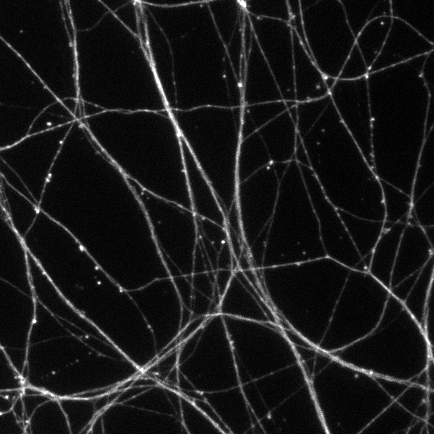

Supplement: Supplementary file 3 — Source data Fig. 2 [file 44319_2025_402_MOESM3_ESM.zip › Figure 2/2C/Aim2_maint_crop.tif]

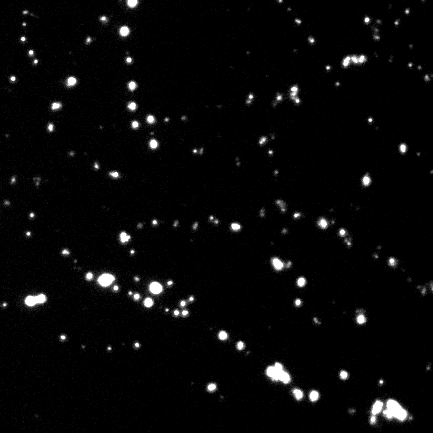

Supplement: Supplementary file 3 — Source data Fig. 2 [file 44319_2025_402_MOESM3_ESM.zip › Figure 2/2C/ASC_global_crop.tif]

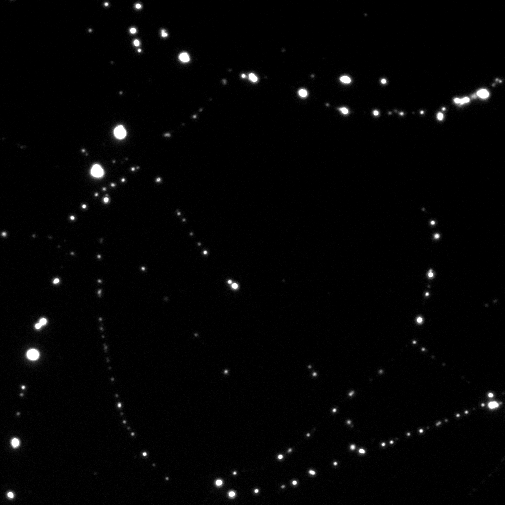

Supplement: Supplementary file 3 — Source data Fig. 2 [file 44319_2025_402_MOESM3_ESM.zip › Figure 2/2C/ASC_local_crop.tif]

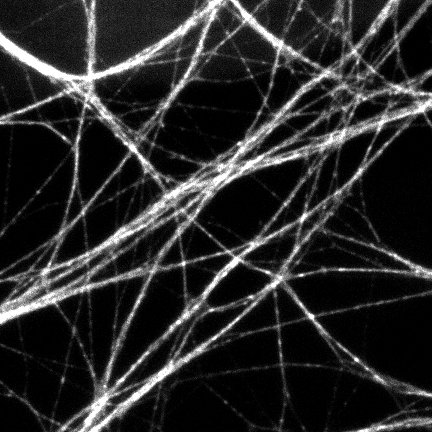

Supplement: Supplementary file 3 — Source data Fig. 2 [file 44319_2025_402_MOESM3_ESM.zip › Figure 2/2C/ASC_maint_crop.tif]

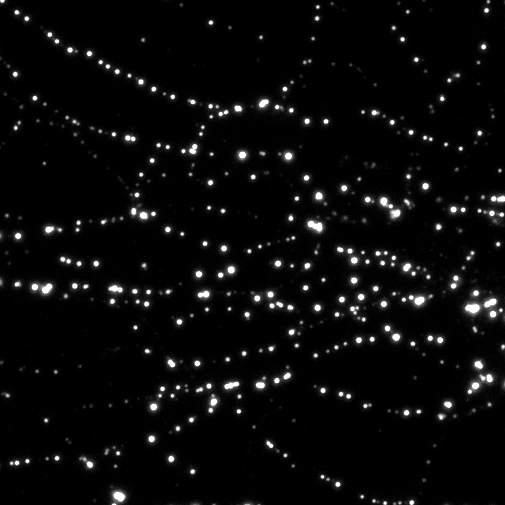

Supplement: Supplementary file 3 — Source data Fig. 2 [file 44319_2025_402_MOESM3_ESM.zip › Figure 2/2C/B6_global_crop.tif]

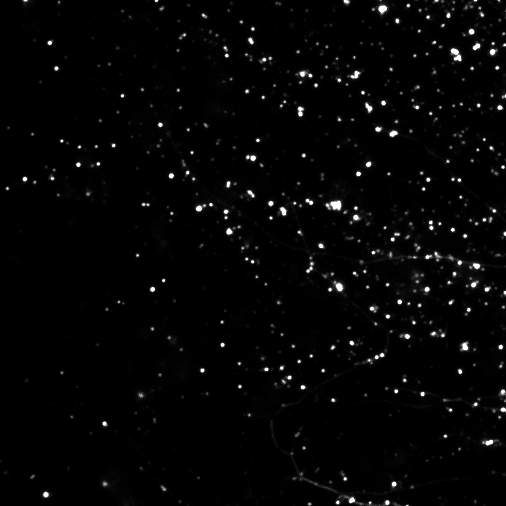

Supplement: Supplementary file 3 — Source data Fig. 2 [file 44319_2025_402_MOESM3_ESM.zip › Figure 2/2C/B6_local_crop.tif]

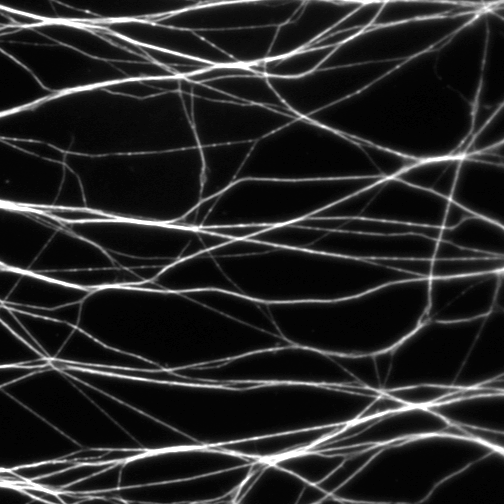

Supplement: Supplementary file 3 — Source data Fig. 2 [file 44319_2025_402_MOESM3_ESM.zip › Figure 2/2C/B6_maint_crop.tif]

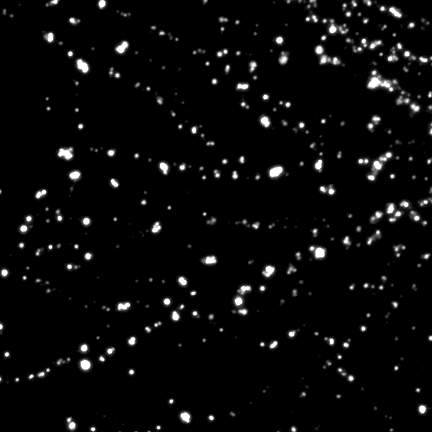

Supplement: Supplementary file 3 — Source data Fig. 2 [file 44319_2025_402_MOESM3_ESM.zip › Figure 2/2C/NLRP3_global_crop.tif]

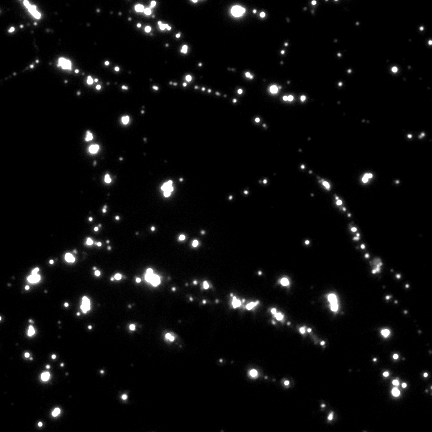

Supplement: Supplementary file 3 — Source data Fig. 2 [file 44319_2025_402_MOESM3_ESM.zip › Figure 2/2C/NLRP3_local_crop.tif]

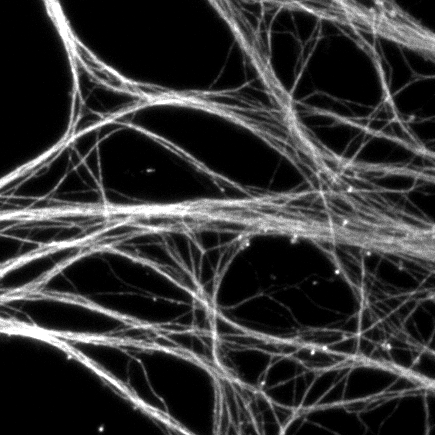

Supplement: Supplementary file 3 — Source data Fig. 2 [file 44319_2025_402_MOESM3_ESM.zip › Figure 2/2C/NLRP3_maint_crop.tif]

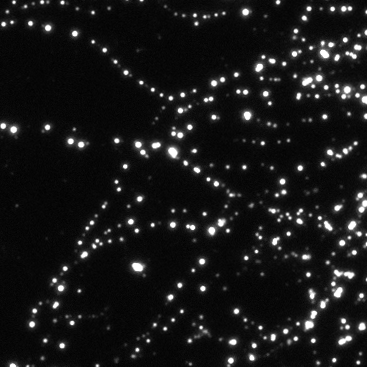

Supplement: Supplementary file 3 — Source data Fig. 2 [file 44319_2025_402_MOESM3_ESM.zip › Figure 2/2E/NLRP1 WT_global_crop.tif]

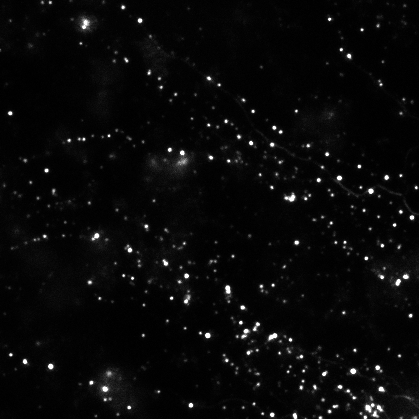

Supplement: Supplementary file 3 — Source data Fig. 2 [file 44319_2025_402_MOESM3_ESM.zip › Figure 2/2E/NLRP1 WT_local_crop.tif]

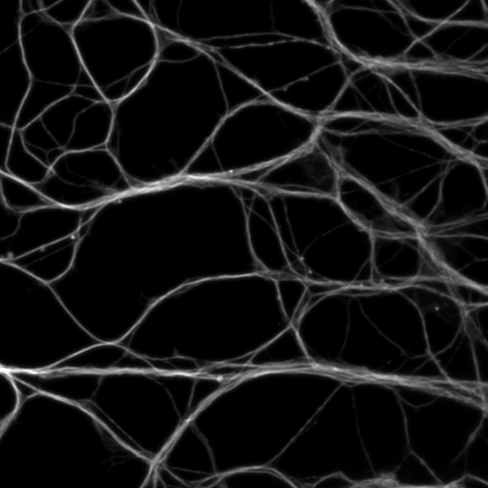

Supplement: Supplementary file 3 — Source data Fig. 2 [file 44319_2025_402_MOESM3_ESM.zip › Figure 2/2E/NLRP1 WT_maint_crop.tif]

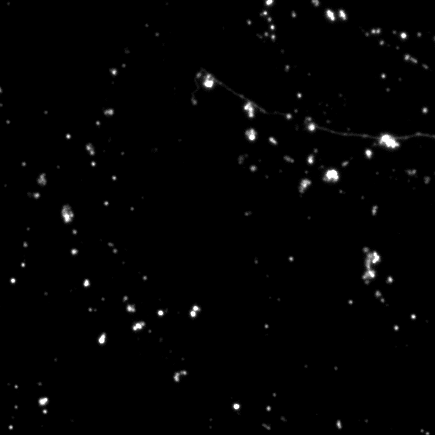

Supplement: Supplementary file 3 — Source data Fig. 2 [file 44319_2025_402_MOESM3_ESM.zip › Figure 2/2E/NLRP1_global_crop.tif]

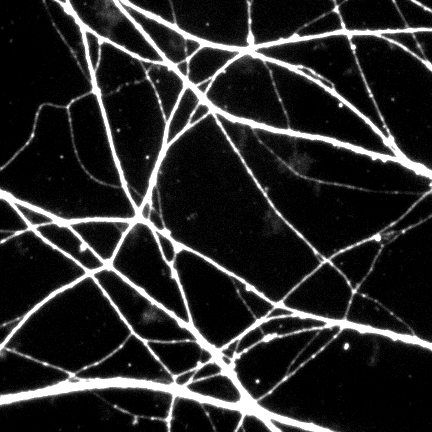

Supplement: Supplementary file 3 — Source data Fig. 2 [file 44319_2025_402_MOESM3_ESM.zip › Figure 2/2E/NLRP1_local_crop.tif]

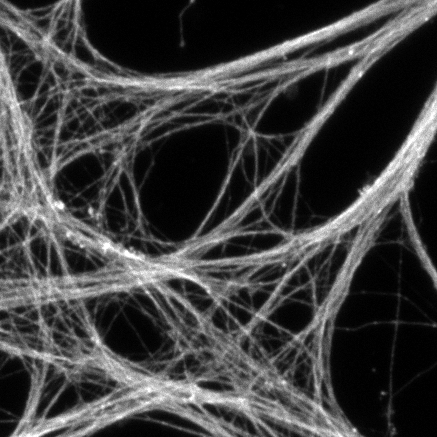

Supplement: Supplementary file 3 — Source data Fig. 2 [file 44319_2025_402_MOESM3_ESM.zip › Figure 2/2E/NLRP1_maint_crop.tif]

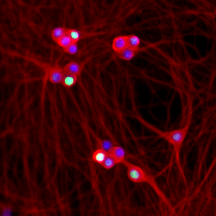

Supplement: Supplementary file 4 — Source data Fig. 3 [file 44319_2025_402_MOESM4_ESM.zip › Figure 3/3A/NLRP1_local_crop_overlay.tif]

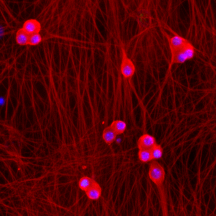

Supplement: Supplementary file 4 — Source data Fig. 3 [file 44319_2025_402_MOESM4_ESM.zip › Figure 3/3A/NLRP1_UNT_crop_overlay.tif]

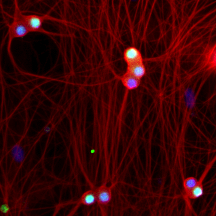

Supplement: Supplementary file 4 — Source data Fig. 3 [file 44319_2025_402_MOESM4_ESM.zip › Figure 3/3A/WT_Local_crop_overlay.tif]

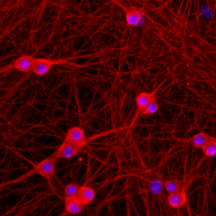

Supplement: Supplementary file 4 — Source data Fig. 3 [file 44319_2025_402_MOESM4_ESM.zip › Figure 3/3A/WT_UNT_crop_overlay.tif]

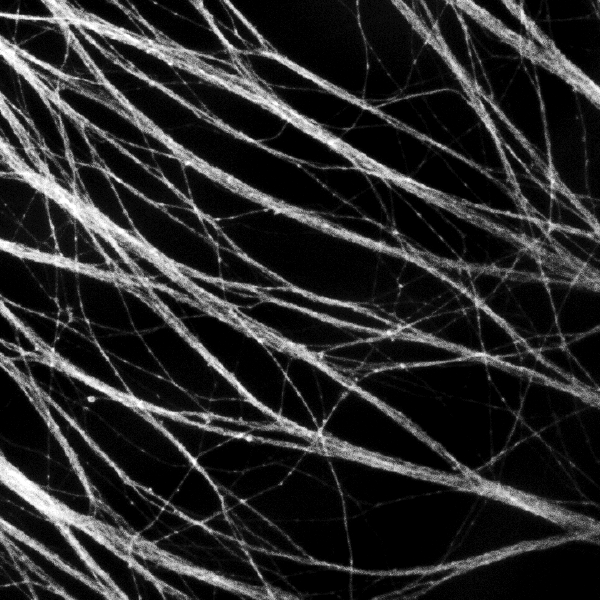

Supplement: Supplementary file 5 — Source data Fig. 4 [file 44319_2025_402_MOESM5_ESM.zip › Figure 4/4A/NGF Maint_10uM VbP_crop.tif]

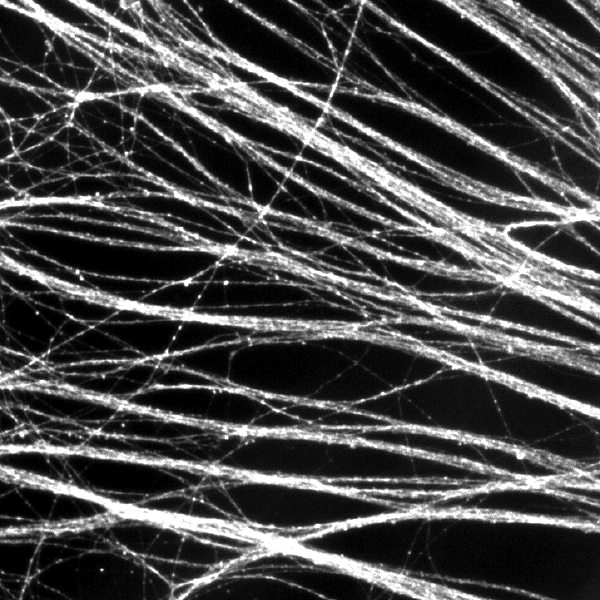

Supplement: Supplementary file 5 — Source data Fig. 4 [file 44319_2025_402_MOESM5_ESM.zip › Figure 4/4A/NGF Maint_VbP figure_crop.tif]

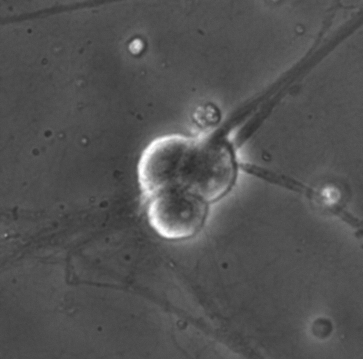

Supplement: Supplementary file 5 — Source data Fig. 4 [file 44319_2025_402_MOESM5_ESM.zip › Figure 4/4C/UPA-CARD_DOX_GFP.tif]

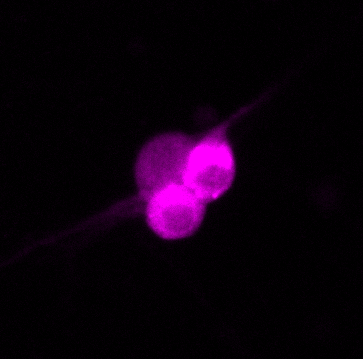

Supplement: Supplementary file 5 — Source data Fig. 4 [file 44319_2025_402_MOESM5_ESM.zip › Figure 4/4C/UPA-CARD_DOX_HA.tif]

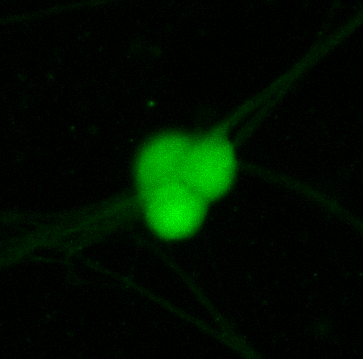

Supplement: Supplementary file 5 — Source data Fig. 4 [file 44319_2025_402_MOESM5_ESM.zip › Figure 4/4C/UPA-CARD_DOX_phase.tif]

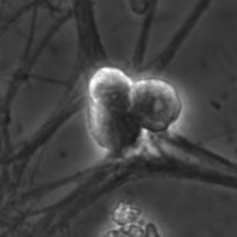

Supplement: Supplementary file 5 — Source data Fig. 4 [file 44319_2025_402_MOESM5_ESM.zip › Figure 4/4C/UPA-CARD_NO DOX_GFP.tif]

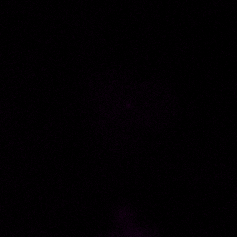

Supplement: Supplementary file 5 — Source data Fig. 4 [file 44319_2025_402_MOESM5_ESM.zip › Figure 4/4C/UPA-CARD_NO DOX_HA.tif]

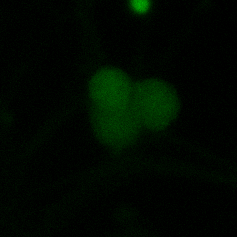

Supplement: Supplementary file 5 — Source data Fig. 4 [file 44319_2025_402_MOESM5_ESM.zip › Figure 4/4C/UPA-CARD_NO DOX_phase.tif]

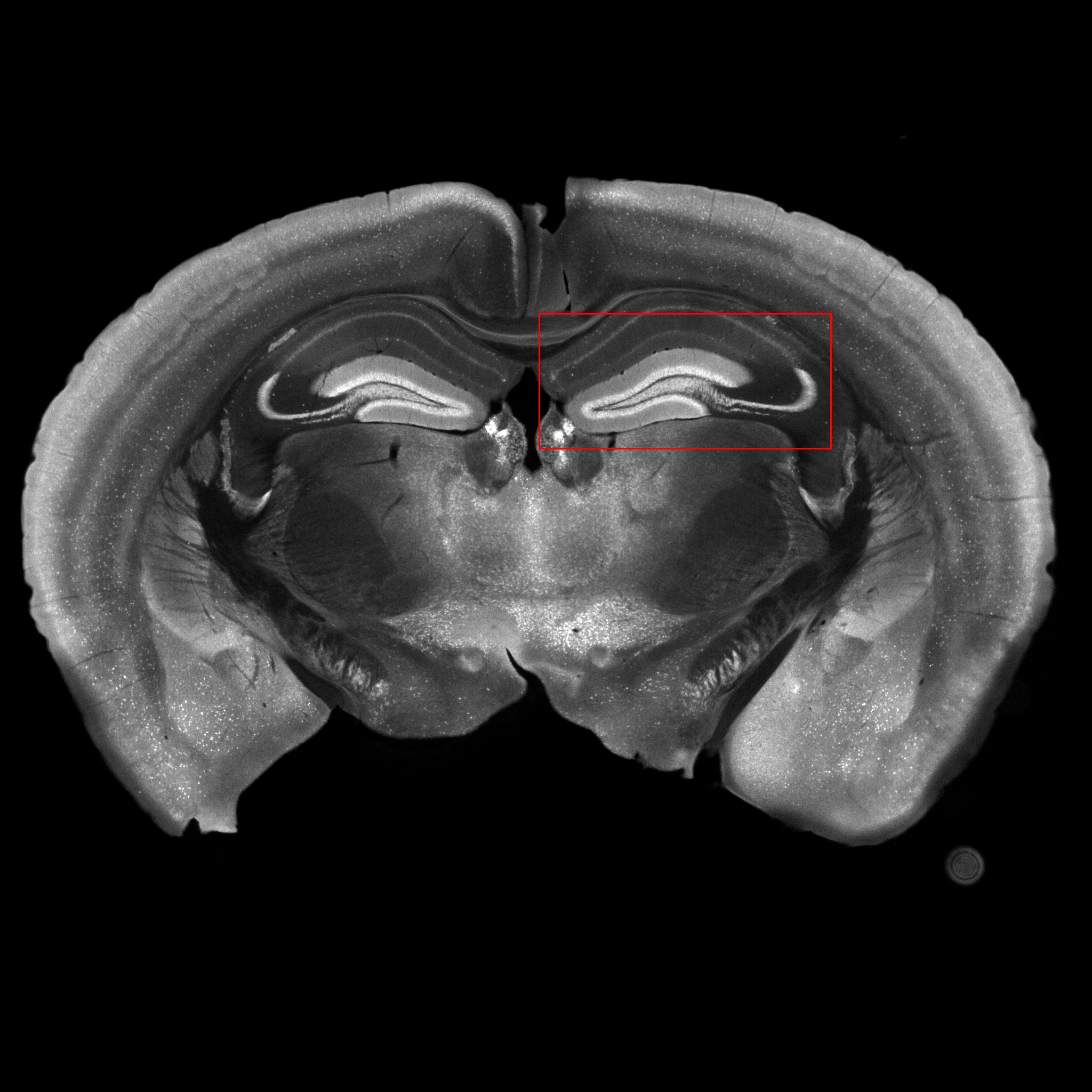

Supplement: Supplementary file 6 — Source data Fig. 5 [file 44319_2025_402_MOESM6_ESM.zip › Figure 5/5A/whole brain and crop window.tif]
