# Supplementary material for: The NLRP1 inflammasome is an essential and selective mediator of axon pruning in neurons
Source: EMBO Rep. 2025 Feb 26;26(7):1724–36. doi: 10.1038/s44319-025-00402-y (PMC11977209; doi:10.1038/s44319-025-00402-y)
Supplement: Supplementary file 8 — Expanded View Figures [file 44319_2025_402_MOESM8_ESM.pdf]

Expanded View Figure

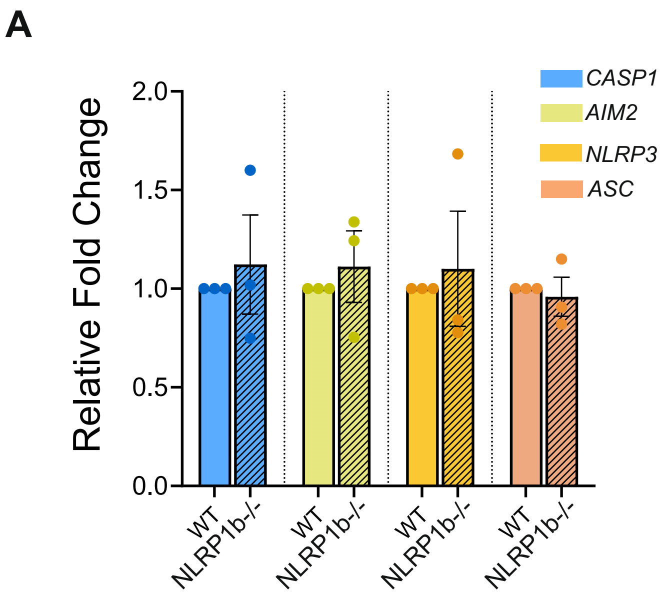

Figure EV1. Inflammasome components remain intact in the NLRP1-deficient sympathetic neurons.

(**A**) Fold change expression of *CASP1*, *AIM2*, *NLRP3*, and *ASC* in wild-type and NLRP1b-deficient sympathetic neurons, normalized to wild-type. Individual data points and mean with standard error of mean represented. Statistical differences were examined by unpaired Student's *t* test ($n = 3$ biological replicates). Source data are available online for this figure.
